# TrialR: critical enablers and the need for reusable Rare Disease Clinical Trial infrastructure in Western Australia

**DOI:** 10.1186/s13023-026-04271-z

**Published:** 2026-04-07

**Authors:** Bradley MacDonald, Maddison Burmaz, Sue Baker, Kaila Stevens, Amanda Newell, Gareth Baynam

**Affiliations:** 1 Rare Care Centre, Perth Children’s Hospital, 15 Hospital Ave, Nedlands, WA 6008 Australia; 2 Curtin Medical School and Curtin Health Innovation Research Institute, Curtin University, Perth, Western Australia Australia; 3 Undiagnosed Diseases Program -WA, Genetic Services of WA, King Edward Memorial Hospital, Subiaco, WA Australia; 4 Western Australian Register of Developmental Anomalies, King Edward Memorial Hospital, Subiaco, WA Australia; 5 Cardiovascular Epidemiology Research Centre, School of Population and Global Health, University of Western Australia, Crawley, WA Australia; 6 Rural Clinical School of Western Australia, School of Medicine, University of Western Australia, Crawley, WA Australia; 7 Wesfarmers Centre of Vaccines and Infectious Diseases, The Kids Research Institute Australia, Nedlands, WA Australia

**Keywords:** Rare diseases, Adaptive platform clinical trials, Critical enablers, Trial access

Over 400 million people globally live with one of over 10,000 different Rare Diseases (RD), making them relatively common when they are considered as a collective [1] RD are recognized as a global health priority, due to the significant physical, emotional and financial hardship experienced [2]. As such, there is significant financial and personal cost to individuals, families and healthcare systems [2]. There are more RD recognized, with most diagnosed in children, as diagnostic techniques improve but with continue poor outcome despite this [1–3]. Fortunately, advances in individualized therapeutics means potential treatments are becoming available for RD for which there was previously no definitive treatment [4, 5] Inequitable access to these therapeutics via clinical trials remains an ongoing issue for children with RD [1]. TrialR is a program to identify critical enablers in Rare Disease Clinical Trials (RDCT) access. Here, we introduce TrialR and discuss the need for access to life-changing trials and the elements of scalable reusable infrastructure necessary for RD trials in Western Australia.

Many of the 63,000 children with RD in Western Australia (WA) are eligible for a clinical trial somewhere. The Rare Care Centre (RCC) in WA, which provides cross sector co-ordination of care for patients with RD, continues to identify numerous eligible patients for clinical trials [6]. Few children seen by the RCC are accessing the potential benefits of RDCT due to trial site location, financial cost and awareness [7, 8] Our platform TrialR delivers a sustainable and scalable increase to RDCT access in WA, whilst facilitating national and international connectivity. We undertook local and international scoping as the first initiative of TrialR with multistakeholder engagement to identify barriers and enablers of RDCT access for patients. A range of themes in RDCT access emerged regarding the state of play for RDCT in WA. We discovered that often, when a trial was accessed by a child in WA, it occurred outside of WA and the family was bearing an associated cost for travel and personal cost (Fig. 1) that was unsustainable [5, 9]. TrialR identifies key stakeholders and advisory groups that want clinical trial access whilst highlighting the critical enablers for facilitating local RDCT access.

**Fig. 1:**
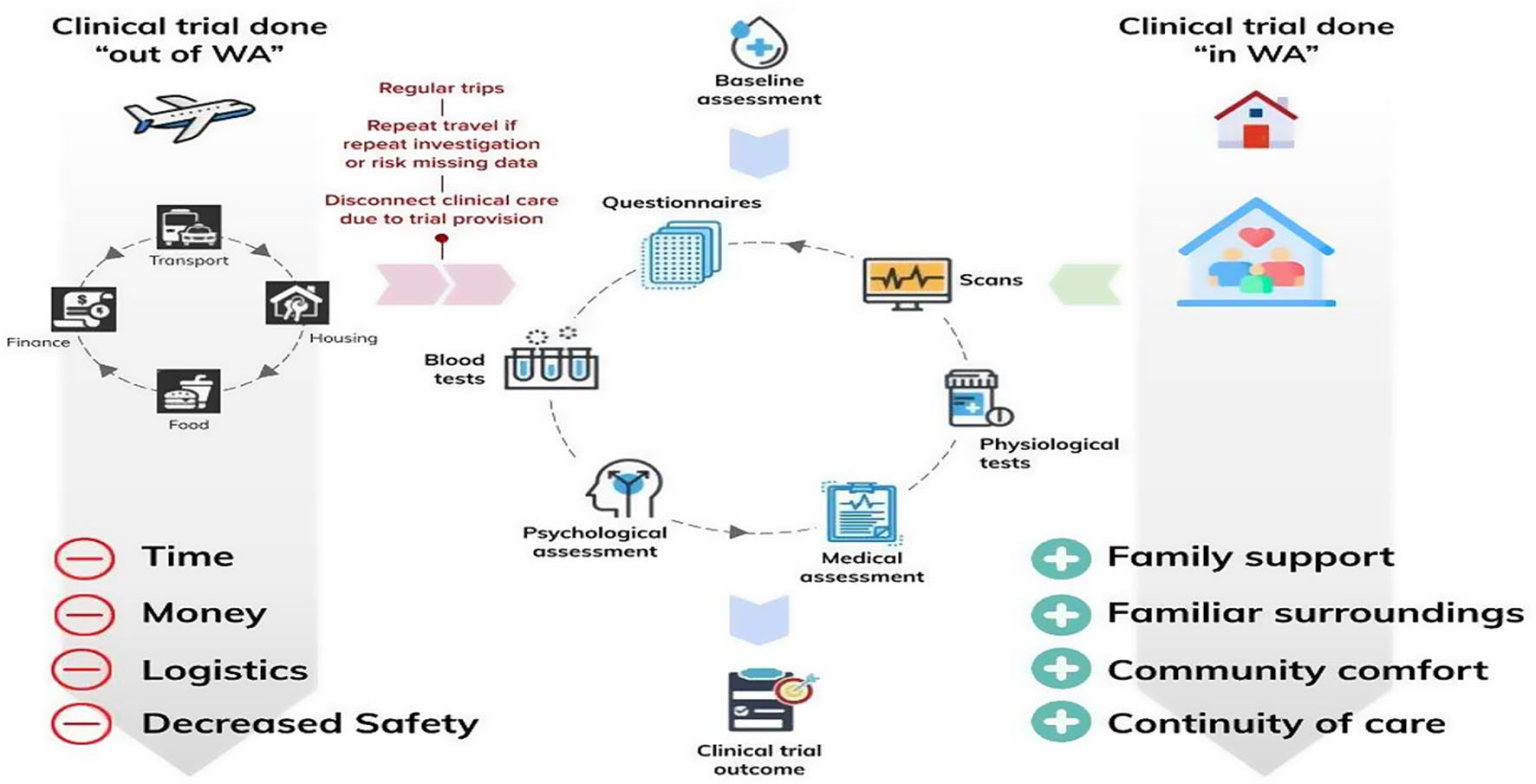
Comparison of a family seeking Rare Disease Clinical Trial access outside of Western Australia compared to the benefits of trial access within the state

At present, WA clinicians are easily deterred from clinical trial involvement due to multiple factors including perceived lack of support, limited experience and lack of resources [10, 11]. Additionally, many clinicians do not have the time to create the unique trial infrastructure or institutional support required for sustaining an individual clinical trial [12]. Due to this, clinicians tended to be reluctant to engage when approached by an industry partner [13]. Industry partners more likely to approach trial sites outside of WA that have had historical trial success and future contact regarding prospective trial opportunities is impacted. Local clinician expertise is subsequently often lost to other trial sites [14]. RDCT support with established infrastructure would support trial bids, develop local expertise, support clinicians and attract future RDCTs to WA.

Seamless and refined RDCT infrastructure and processes can combat the location-based barriers to developing trials. Industry partners more likely to apply their RDCT to larger sites where there are likely more eligible trial patients with a tendency to overlook jurisdictions with smaller populations like WA (~ 2.6 Million people) [15]. As industry partners also want trial sites that have the expertise to quickly get a trial up and running successfully; smaller sites need to focus on expertise with the capability to quickly get a trial up and running successfully [16]. Infrastructure to run RDCTs in an efficient manner in WA is therefore critical to overcome the limitations of our geographical location and size. TrialR identifies many key factors that make quick trial access in WA a possibility that would attract future RDCT.

Multiple components of RDCT infrastructure need development in WA, with a focus on making them reusable. Adaptive platform trials may add regulatory efficiency to this process and allow scalability of individual trials over time and streamlines unique RDCT requirements including complex protocols specific to therapy and disease [17–20]. Multiple key aspects of RDCT remain similar across different trials for various RD. For example, many trials looking at efficacy of therapies also require periodic reviews using developmental assessments. Exploiting RDCT similarities, such as the need for repeated developmental assessments, would create a reusable process for RDCTs that is critical for trial efficiency and value [21]. TrialR highlights the processes that create inefficiencies in RDCTs in WA so the future maintenance of RDCT infrastructure is reusable.

Our current ethical and regulatory approval pathways can delay local approval for RDCTs and make them unfeasible. At present, every RDCT has its own individual approvals and processes to proceed. The trial protocol, provided by overseeing industry partners, is often amended to the local regulatory formatting [22]. If there are issues with this process, the trial may not be approved or the length of time to obtain approval means other sites may ‘win’ the trial over WA [23]. We recognized that previous trials within our system succeeded due to passionate and forward-thinking individual clinicians devoting their own time for trial success. Clinicians though, can reach capacity quickly and be deterred by the time-consuming processes associated with regulatory approval in our current system [22]. TrialR refines quick and scalable ethical and regulatory approval pathways for building capacity for simultaneous RDCT.

Trial successes increase capacity for more trial successes. RDCT success impacts local attitudes towards clinical trials, promotes funding and builds our recognition as a paediatric trial site [24]. Cultural shift and acceptance of clinical trials as treatment starts with successful and easily instigated clinical trials. Expertise gained by clinicians can then be shared with others to promote their involvement [13]. Funding for clinical trials can provide a resource for infrastructure that would be valuable to maintain across other trials where applicable [25]. TrialR identified many critical enablers for trial success that need to be leveraged for RDCTs. TrialR is the beginning of a range of practical changes to start routine RDCTs in WA, leaving legacy infrastructure to perpetuate trial completion.

The TrialR initiative provides the impetus for increasing the availability of RDCTs in WA. The TrialR scoping review notes significant critical enablers for RDCT access. Learning from this process led to the TrialR Advisory Committee to further inform direction for building RDCT access [26, 27]. The advisory committee, formed of consumers and clinicians, informs the direction of TrialR, promotes key enablers which require early implementation. We added a scheme to support clinical staff conducting trials and emphasized protected clinical time via financial aid and nursing support [28, 29]. Regulatory applications for an adaptive clinical trial for RD are now underway with plans to use this as core regulatory infrastructure for trials [30, 31].

TrialR progresses infrastructure for RDCT access through a trials as treatment approach. We continue to identify key critical enablers for reusable scalable local clinical trial infrastructure. At present, there are interested industry partners that have therapeutics they wish to trial, but no way to find a principal investigator, team or expertise in WA for RDCT implementation. The TrialR initiative dismantles barriers for RDCT with a future adaptive platform trial approach and streamlined ethics process. We are creating a hub for principal investigators and industry partners’ collaboration; where cutting-edge trials can be implemented by supplementing principal investigator time. TrialR connects world-class infrastructure, cutting-edge facilities and committed stakeholders in pediatric healthcare. The TrialR roadmap for infrastructure development and clinical trial implementation continues to attract industry partners to implement trials within our jurisdiction. As such, our service will become accountable to RD patients for trial access and provide an opportunity for other centers to replicate the same initiative.

## Data Availability

Not applicable.
